# Solvent Casting and UV Photocuring for Easy and Safe Fabrication of Nanocomposite Film Dressings

**DOI:** 10.3390/molecules27092959

**Published:** 2022-05-05

**Authors:** Laura Di Muzio, Prisca Simonetti, Vito Cosimo Carriero, Chiara Brandelli, Jordan Trilli, Claudia Sergi, Jacopo Tirillò, Francesco Cairone, Stefania Cesa, Giulia Radocchia, Serena Schippa, Stefania Petralito, Patrizia Paolicelli, Maria Antonietta Casadei

**Affiliations:** 1 Department of Drug Chemistry and Technologies, Sapienza University of Rome, 00185 Rome, Italy; simonetti.1748558@studenti.uniroma1.it (P.S.); vitocosimo.carriero@uniroma1.it (V.C.C.); chiara.brandelli@uniroma1.it (C.B.); jordan.trilli@uniroma1.it (J.T.); francesco.cairone@uniroma1.it (F.C.); claudia.sergi@uniroma1.it (S.C.); stefania.petralito@uniroma1.it (S.P.); mariaantonietta.casadei@uniroma1.it (M.A.C.); 2 Department of Chemical Engineering Materials Environment, Sapienza University of Rome, 00184 Rome, Italy; stefania.cesa@uniroma1.it (C.S.); jacopo.tirillo@uniroma1.it (J.T.); 3 Department of Public Health and Infection Disease, Microbiology Section, Sapienza University of Rome, 00185 Rome, Italy; giulia.radocchia@uniroma1.it (G.R.); serena.schippa@uniroma1.it (S.S.)

**Keywords:** thin film, silver nanoparticles, gellan gum methacrylate, solvent casting, photocuring, antimicrobial activity, colorimetry, wound dressings

## Abstract

The aim of this work was to optimize and characterize nanocomposite films based on gellan gum methacrylate (GG-MA) and silver nanoparticles (AgNPs) for application in the field of wound dressing. The films were produced using the solvent casting technique coupled with a photocuring process. The UV irradiation of GG-MA solutions containing glycerol as a plasticizer and different amounts of silver nitrate resulted in the concurrent crosslinking of the photocurable polymer and a reduction of Ag ions with consequent in situ generation of AgNPs. In the first part of the work, the composition of the films was optimized, varying the concentration of the different components, the GG-MA/glycerol and GG-MA/silver nitrate weight ratios as well as the volume of the film-forming mixture. Rheological analyses were performed on the starting solutions, whereas the obtained films were characterized for their mechanical properties. Colorimetric analyses and swelling studies were also performed in order to determine the AgNPs release and the water uptake capacity of the films. Finally, microbiological tests were carried out to evaluate the antimicrobial efficacy of the optimized films, in order to demonstrate their possible application as dressings for the treatment of infected hard-to-heal wounds, which is a demanding task for public healthcare.

## 1. Introduction

Human skin, working as a boundary with the external environment, is continuously exposed to multiple injuries [1]. Physical or thermal damage can cause defects or interruptions in the epidermis of the skin, forming a wound. The healing process of a wound is influenced by several factors, including size, depth, degree of injury [2] and its eventual contamination. For these reasons, skin lesions heal at different times and with different outcomes [3,4]. In particular, acute wounds usually recover in a short period of time, restoring normal anatomical structure and function. On the contrary, the repair of chronic wounds is challenging, particularly when it is complicated by the appearance of infections during the healing process [5,6]. The onset of chronic wounds can lead to a net decrease in patients’ quality of life and also produces a considerable expense for the health system.

The most common wound infections are those arising from abrasions and surgical wounds (one of the most common causes of health-care-related infections that can occur after an invasive surgical procedure) and are caused by bacteria [7]. Among the main microorganisms responsible for wound infections are cocci, gram-positive bacteria such as *Staphylococcus aureus*, coagulase-negative Staphylococci and Enterococci including the most dangerous methicillin-resistant *S. aureus* (MRSA) and vancomycin-resistant *S. aureus* (VRSA). The presence of antibiotic resistant bacteria, often biofilm producers, makes the healing of infected wounds more difficult and challenging and compromises patients’ lives [8]. Therefore, the search for new effective treatments that prevent and counteract infections, in addition to promoting the wound healing process, has increased in recent years [9,10]. Wound dressings are generally curative materials easily adaptable to different anatomical regions and endowed with high strength and elasticity [11,12,13]. In this scenario, systems based on hydrogels have received attention in recent years in therapeutically advanced wound care management, since they are particularly effective for the scope [14,15,16]. Hydrogels can be defined as highly hydrated polymeric materials whose structural integrity is ensured by physical or chemical inter- and intramolecular crosslinks between polymer chains. This feature makes these polymer networks capable of absorbing wound exudate and maintaining a moist environment, allowing gas exchange and thermal insulation, which are essential characteristics that support hydrogels in promoting repair and reversing chronic wounds. They are generally safe and can be easily and painlessly removed from the injury surface. Moreover, hydrogels possess adequate biomechanical and viscoelastic properties for suturing to the wound surface or applying to it. Globally, hydrogels meet most of the criteria for modern wound dressings, especially when produced in the form of films [17,18]. Wound dressing films are particularly convenient for wound care management because they are flexible, easy to apply, provide a physical barrier to prevent external opportunistic bacteria’s entry and enable easy inspection of the injured site, without the need for dressing removal. They also allow for the regulation of gas exchange and the uptake of the exudate through the swelling process. Furthermore, the components of the films can actively participate in promoting the healing process [19,20]. Even more, wound dressing films can be loaded with active substances, including organic or inorganic nano-sized materials, capable of preventing and eradicating infections and offering better control over drug concentration for long times and improved drug stability at the wound site [21]. The wide application of nanomaterials in pharmaceutical and biomedical fields stems from the dramatic improvements of their chemical, physical, mechanical and optical properties in comparison to bulk materials. In this regard, metal-based nanoparticles (NPs) demonstrated great effectiveness both as nano-biocides, due to their intrinsic and broad-spectrum antimicrobial activity [22], and as nano-carriers for the delivery of antimicrobial drugs [23]. The research on metal-based NPs has heavily increased in recent years, and it is not expected to decrease due to their well-described antimicrobial activity against both gram-positive and gram-negative bacteria. Among all the investigated metal-based NPs, those made of silver (silver nanoparticles, AgNPs) are the most widely studied nowadays. The antibacterial and antioxidant activity, as well as the efficacy of silver in the treatment of wounds, have been well-known for a long time, and even though its use was abandoned when antibiotics were discovered, nowadays, silver is once again receiving significant attention as an alternative to traditional antibiotics to overcome bacteria resistance issues [24,25]. In this scenario, silver nanotechnology has led to increased demand for its medical application, including the control of infections in wound dressings. Indeed, AgNPs are some of the most intriguing and extensively employed materials for wound healing because of their excellent antimicrobial properties. Although the mechanism of action has not been fully understood, many articles highlight the therapeutic potential of AgNPs embedded in polymeric networks and their role in controlling wound contamination and biofilm formation [26,27,28,29,30,31]. AgNPs loaded wound dressings can overcome the problems of silver ions and silver sufadiazine (SSD) containing wound dressings, such as their rapid inactivation, unsustainable antibacterial nature, low biocompatibility and poor stability [30]. Moreover, the incorporation of AgNPs within polymer networks can improve their therapeutic potential [32]. Indeed, AgNPs generally tend to aggregate into larger particles to decrease the available surface area, thus minimizing their interfacial free energy. In this way, the surface area to volume ratio decreases, and, consequently, their antimicrobial activity reduces, because the smaller specific surface area decreases the binding ability of AgNPs to the bacterial cell. Furthermore, AgNPs aggregation brings about a slower release rate of silver ions, which are conducive to the antimicrobial activity [33,34]. Therefore, nano-silver wound dressings show great prospects in wound repair treatment and are already widely used in the treatment of chronic wounds, such as bacterial infected and purulent wounds. Although nano-silver containing dressings show clear advantages when applied to infected wounds, some problems have been identified that seriously inhibit their wide applications, such as the easy agglomeration of AgNPs and the toxicity during preparation. Generally, there are two ways to load AgNPs into wound dressings. The first one consists of directly blending or soaking preformed AgNPs into dressings. In the second way, the dressing is generally first fabricated, while, in a second step, the matrix material is soaked with silver ions. Silver is then reduced in situ to obtain the final nano-silver dressing. In both cases, AgNPs are chemically synthesized, using strong reducing reagents, such as hydrazine and sodium borohydride, which are toxic for cells. The synthesis of high purity AgNPs is still a challenge, and there is a continuous demand for improved and safe synthetic techniques [35,36]. In this scenario, UV irradiation of silver salts solutions is considered one of the most rapid, safe and efficient methods [37,38]. Starting from this evidence, the aim of this work was the optimization and characterization of nanocomposite thin films containing colloidal silver (AgNPs) for application in the field of wound dressings. The nanocomposite films were produced using the solvent casting technique coupled with UV photocuring. The starting materials used for the preparation of the films were gellan gum (GG), a natural and biocompatible polysaccharide, and glycerol (Gly) employed as a plasticizer. Despite the well-known film-forming features of GG, its application for the development of antimicrobial wound dressings has barely been investigated [39]. GG and Gly were chosen based on recently reported results, demonstrating that they can be successfully combined to produce polymeric thin films with adequate physical and mechanical properties [40]. Considering that we decided to use UV irradiation for AgNPs synthesis, GG was chemically modified by the reaction with methacrylic anhydride to produce a methacrylate derivative of GG (GG-MA), due to its ability to form chemical and biocompatible hydrogels under UV irradiation [41,42]. Therefore, the solvent casting technique was coupled with UV photocuring to allow for the one-step production of nanocomposites films. Indeed, the photocuring of GG-MA solutions, containing the plasticizer and different concentrations of AgNO_3_, should result in the simultaneous formation of the polymeric matrix and the in situ generation of AgNPs through the reduction of Ag^+^ ions to Ag^0^. At first, the composition of the films was optimized, varying the concentration and weight ratio of GG-MA and Gly, as well as the volume of the casted solution. Rheological analyses were performed on the different film-forming mixtures in order to evaluate their suitability for the solvent casting process. The dried films were subjected to physical and mechanical characterization, including thickness measurements, tensile studies and swelling evaluations. Colorimetric analyses were performed in order to verify the correlation between color change and the reduction of Ag^+^ ions to Ag^0^ and to assess the release of AgNPs. Finally, biological tests allowed for the assessment of the antimicrobial efficacy of AgNPs-loaded films in order to verify their possible application as dressing materials for the management of infected wounds. The proposed procedure can represent an interesting approach for the facile and safe fabrication of nanocomposite film dressings.

## 2. Materials and Methods

### 2.1. Materials

Gellan gum (Gelzan), glycerol (Gly), methacrylic anhydride (MAA), triethylamine (TEA), anhydrous dimethyl sulfoxide (DMSO), deuterated water (D_2_O), 4-dimethylaminopyridine (4-DMAP), phosphate buffer saline (10 mM phosphate and 150 mM NaCl, pH = 7.4, which was labelled PBS), polyethylenglycole (PEG 20,000 Da), 2-hydroxy-4-(2-hydroxyethoxy)-2-methyl-propiophenone (Irgacure 2959) and dialysis tubes (cut-off 12–14 kDa) were purchased from Sigma Aldrich (Milan, Italy). Silver nitrate (AgNO_3_), monobasic potassium phosphate (KH_2_PO_4_, used to prepare 40 mM phosphate buffer, pH = 7.4, which was labelled PB), sodium hydroxide (NaOH) and hydrochloric acid (HCl) were purchased from Carlo Erba (Milan, Italy). Finally, 1-methyl-2-pyrrolidinone from Fluka (St. Louis, MO, USA). Triptone Soy Agar (TSA) and Brain Heart Infusion broth (BHI) were purchased from Oxoid (Basingstoke, UK).

### 2.2. Synthesis of Gellan Gum Methacrylate (GG-MA)

The functionalization of GG was performed as reported in the literature [41]. The procedure was slightly modified to allow for faster and easier dissolution of the polymer. Specifically, GG (1 g) was solubilized in anhydrous DMSO (65 mL) at 90 °C for 3 h under magnetic stirring. After complete solubilization, the temperature of the polymer solution was brought to 60 °C, and, subsequently, 4-DMAP (0.04 g), TEA (0.5 mL) and MAA (0.5 mL) were added. The reaction mixture was kept under magnetic stirring at 60 °C for 24 h. The synthesized polymer was purified by dialysis against distilled water and freeze-dried, employing a freeze-drier LIO5P (5 Pascal, Trezzano sul Naviglio, Italy). The polymeric derivative was characterized by ^1^H-NMR analysis in D_2_O and recorded with a Bruker AC-400 spectrometer (Bruker Corporation, Billerica, MA, USA). The degree of derivatization (DD%) was calculated as the ratio of the area of the protons of the methyl signal of the methacrylic group (at 1.9 ppm) and the methyl signal of rhamnose of the repetitive unit of GG (at 1.3 ppm), multiplied by 100. In this way, a DD% of 100 ± 1 was obtained. The small changes made to the synthesis protocol allowed for the obtention of a more efficient and reproducible methacrylation degree of GG-MA.

### 2.3. Rheological Characterization

The rheological measurements were conducted using a Discovery TA HR-1 stress-control rheometer (TA Instruments, New Castle, DE, USA). A cone-plate geometry with a diameter of 40 mm (α 1.005°, gap 27 µm) was used for all the experiments [40]. Solutions of GG (2.0% *w/v*) and of GG-MA containing different concentrations of Gly (2.0 and 4.0% *w/v*) were prepared, solubilizing both the components in distilled water at 50 ± 0.1 °C for 30 min under magnetic stirring. Then, 0.5 mL of the obtained solutions were poured on the Peltier plate of the rheometer, pre-heated at 50 °C. Oscillatory temperature-sweep analysis was carried out by decreasing the temperature from 50.0 to 30.0 ± 0.1 °C (cooling rate 3.0 ± 0.1 °C/min), keeping a constant frequency of 1 Hz and a deformation of 1%, which was previously determined using oscillatory strain-sweep tests. In addition, flow-sweep measurements were carried out on GG-MA solutions (2.0% *w/v*) containing or not containing different concentrations of Gly (2.0, 3.0 and 4.0% *w/v*). In particular, the viscosity as a function of the shear rate was measured, applying shear stresses in the range of 0.0005–500 Pa. The flow-sweep measurements were carried out at 40.0 ± 0.1 °C, the same temperature employed during the casting process. All the experiments were carried out at least in triplicate.

### 2.4. Film Preparation

GG-MA based films were produced using the solvent casting technique [40] coupled with a photo-polymerization process induced by UV light in the presence of the photoinitiator irgacure 2959. The films were prepared by dissolving the opportune amount of GG-MA and Gly in distilled water at 50 °C for 1 h under magnetic stirring. After complete solubilization, the temperature was decreased to 40 ± 1 °C, and appropriate volumes of AgNO_3_ solution (30% *w/v*) and 7.5 µL/mL of irgacure 2959 (20% *w/v* solution in *N*-methylpyrrolidone) were added. Then, the film-forming mixture was poured onto a glass support (diameter of 4.5 cm) previously coated with a solution of PEG 20,000 (30% *w/v*) and subjected to irradiation with a UV lamp G.R.E (Helios Italquartz Srl, Cambiago, Italy). 125 W for 20 min. The first film samples were prepared with 6 mL of aqueous mixtures containing 2.0% *w/v* of GG-MA and 3.0% *w/v* of Gly, as already reported in the literature for the preparation of GG-based thin films [40,43]. Different volumes of AgNO_3_ aqueous solution (30% *w/v*) were used in order to produce films containing increasing quantities of AgNPs. At the end of the photocuring step, the samples were heat dried in an oven for 15 h at 40 ± 1 °C. In order to produce films with improved mechanical and physical properties, further samples were prepared by varying the concentration of Gly and GG-MA in the precursor solution or increasing the volume of the casted polymer solution from 6 to 12 mL. In the last case, the drying step was extended to 18 h. All the prepared films and their composition are reported in Table 1. The film samples obtained from film-forming mixtures containing different concentrations of GG-MA and Gly were labelled with different letters, whereas a subscript was used to specify the amount of silver salt included in the formulation.

### 2.5. Film Characterization

Films F_5_, F_10_, F_20_, F_30_ and F_40_ were characterized for their physical and mechanical properties, as described in the following sections.

#### 2.5.1. Measurement of Film Thickness

The thickness of all the prepared films was measured by using the thickness gauge Mitutoyo Digimatic Micrometer (Mitutoyo Corporation, Lainate, Italy). The measurements were carried out in six different points of the same film in order to evaluate the homogeneity of the prepared samples. The results were reported as mean values ± standard deviation.

#### 2.5.2. Scanning Electron Microscopy (SEM) and Energy Dispersive Spectroscopy (EDS)

The films were submerged in 30 mL of phosphate buffer (PB, pH = 7.4) for 5 min to eliminate Gly present in the formulations and then freeze-dried. The resulting samples were morphologically characterized through a field-emission scanning electron microscope (FE-SEM) MIRA 3 (Tescan, Brno, Czech Republic). The morphological characterization was supported by an elemental analysis through the EDS Octane Elect (Edax, Leicester, UK). Samples sputter coating with gold or other conductive materials was intentionally avoided to prevent EDS analysis alterations.

#### 2.5.3. Mechanical Characterization: Tensile Tests

The mechanical performance of the films was evaluated through tensile testing [40]. The tests were performed with a Zwick/Roell Z010 (Zwick/Roell Srl, Genova, Italy) equipped with a 100 N load cell. A test speed of 2 mm/min was used. Specimen length was slightly variable due to the circular nature of the produced films, but a fixed grip-to-grip separation of 15 mm was used, whereas specimen width was 3.5 mm ± 0.3 mm. The experiments were carried out at least in triplicate, and the results were reported as mean values ± standard deviation.

#### 2.5.4. Colorimetric Analysis

The films were subjected to colorimetric analysis using a MetaVue X-Rite TM colorimeter (X-Rite, Grand Rapids, MI, USA), featuring an LED illuminant/45-0°. The L* (luminance), a* (redness-greenness), b* (yellowness-blueness), C*_ab_ (chroma) and h° (hue angle) values were calculated using the iColor software (X-Rite, Grand Rapids, MI, USA). The CIEL*a*b* parameters were used to describe the chromatic properties of the heat dried films characterized by a progressively increasing content of AgNPs (F_5_, F_10_, F_20_, F_30_ and F_40_).

### 2.6. Swelling Studies

The films were characterized through dynamic swelling measurements [40]. Each film was divided into three portions of comparable size, weighed and immersed in 15 mL of PB (pH = 7.4) at 37.0 ± 1.0 °C. At predetermined time points, the samples were extracted from the medium, gently wiped to remove the liquid in excess and weighed. The degree of swelling (*Q*) was calculated as follows:

$$Q=\frac{W_{s}}{W_{d}}$$

where *W_s_* and *W_d_* represent, respectively, the weight of the swollen sample at time *t* and that of the dry sample. The measurements were continued up to 24 h. However, after 15 min, some specimens of each sample were recovered and heat dried at 40 ± 1 °C until constant weight (*W_e_*). These samples were used to evaluate the contribution to weight loss due to the outward diffusion of Gly and AgNPs. In this case, the degree of swelling was expressed as water uptake (*WU*) and calculated as follows:

$$WU=\frac{W_{s}-W_{e}}{W_{e}}$$

The experiments were carried out at least in triplicate, and the results were reported as mean values ± standard deviation.

### 2.7. Release Studies

Silver release was qualitatively monitored by colorimetric analysis and DLS measurements. To this end, F_40_ films were selected for the investigation because they were characterized by the highest silver loading and allowed for a more evident chromatic variation of the release medium. Colorimetric analysis was carried out as reported in Section 2.5.4, whereas dynamic light scattering (DLS) measurements were performed with a Zetasizer Pro (Malvern Panalytical, Malvern, UK) analyzer. The release studies were carried out by immersing the films in 100 mL of PB (pH = 7.4) at 37.0 ± 0.1 °C without stirring. At defined time points, from 1 min to 4 h, aliquots of 1 mL of the release medium were withdrawn and analyzed to evaluate the presence of AgNPs by color evaluation and DLS measurements. At the end of the analyses, the samples taken from the release medium were immediately placed back into the release solution to restore the initial volume. The experiments were carried out in triplicate.

### 2.8. Antimicrobial Activity of Films Loaded with AgNPs

#### 2.8.1. Antimicrobial Activity in Liquid Culture Media

The antimicrobial activity of films loaded with different quantities of AgNPs was tested on two collection strains, *Staphylococcus aureus* ATCC 6538 (American Type Culture Collection, Manassas, VA, USA) and *Escherichia coli* MG1655 (ATCC 700926) (American Type Culture Collection, Manassas, VA, USA). These strains were conserved at −80 °C in glycerol stocks. For the experiments, the strains were cultured directly from glycerol stock on a TSA plate and incubated aerobically overnight at 37 °C. A colony grown on a TSA plate was inoculated in BHI and incubated overnight at 37 °C with agitation (180 rpm). The bacterial suspension concentration was then measured spectrophotometrically at 600 nm (OD600) (BioPhotometer, Eppendorf, Hamburg, Germany). For the assay, both for *E. coli* MG1655 and *S. aureus* ATCC 6538, a bacterial suspension 1 × 10^8^ CFU/mL was seeded in a 24-well plate (500 µL/well) containing a disk of film for each well. The films had all the same dimension (1 cm of diameter) and weight (10 mg c.a.) but were loaded with different concentrations of AgNPs (5, 10, 20, 30, 40 mg/mL). A higher concentration of AgNPs (80 mg/mL) was also tested using two samples of the F_40_ film loaded with 40 mg/mL of AgNPs. For each bacterial strain, a well without the film was used as the positive control of bacterial growth. The plate was then incubated at 37 °C for 24 h. After the incubation, the contents of each well were collected, and serial 10-fold dilutions were prepared in PBS and seeded on TSA plates in order to count the CFU/mL. Each concentration was tested three times. Furthermore, the maintenance of the activity over time was evaluated by performing antimicrobial assays for four days using the same disks of F_40_ nanocomposite film. Briefly, the disk was added to a bacterial suspension 1 × 10^8^ CFU/mL of *E. coli* MG*1655* or *S. aureus* ATCC *6538*, as described above. Every 24 h (time points were 24, 48, 72 and 96 h), the medium was collected and replaced with a fresh bacterial suspension (1 × 10^8^ CFU/mL). The collected media were then used to estimate the bacterial load by seeding on a TSA plate, as described before.

All the experiments were performed in triplicate, and the results were reported as mean values ± standard deviation.

#### 2.8.2. Antimicrobial Activity in Solid Culture Media (Diffusion Test)

To create a more hydrated/humid environment necessary for the diffusion of AgNPs, from the films inside the media, we decided to perform the diffusion tests using double layer plates. A bottom layer was prepared with TSA 1% of agar, and a top layer (a germs agar) was prepared with TSA 0.6% of agar and 1 × 10^8^ CFU/mL of bacteria (*E. coli* MG1655 or *S. aureus* ATCC 6538). Disks of film, all of the same dimension (1 cm of diameter) and weight (10 mg c.a.), loaded with different concentrations of AgNPs (5, 10, 20, 30, 40, 80 mg/mL) were put on plates. Then, the plates were incubated at 37 °C for 24 h. After the incubation, the halos formed around the disks were observed to evaluate if the AgNPs were able to diffuse in the agar medium and inhibit bacterial growth.

### 2.9. Statistical Analysis

The statistical significance was assessed using the GraphPad Prism 9 statistical software package (GraphPad Software, Inc., San Diego, CA, USA), using the one-way ANOVA test for multiple comparison and Dunnett’s multiple comparisons test to compare paired samples. Differences were considered statistically significant when the *p*-value was less than 0.05.

## 3. Results and Discussion

### 3.1. Film Preparation and Optimization

A methacrylic derivative of GG (GG-MA) was employed to develop nanocomposite films containing AgNPs. The use of this photocrosslinkable polymer should allow for the production of a long-lasting and stable polymer network and the concurrent reduction of silver ions in a single step UV-based photocuring process. Solvent casting has already been coupled with UV irradiation for the fabrication of nanocomposite films. UV photocuring has always been used to allow for either polymer crosslinking or silver reduction [44,45]. Instead, in this work, it was used to let the two processes occur in the same fabrication step.

GG-MA was synthesized following a classic and well-established procedure based on the reaction of GG with MAA in anhydrous DMSO [41,42]. The synthesis protocol and the molar ratio of the reagents were optimized to attain a DD% of 100 ± 1, which was calculated by the ^1^H-NMR spectrum as the ratio of the area of the methyl protons of the methacrylic group (at 1.9 ppm) and the methyl signal of rhamnose of the GG repeating unit (at 1.3 ppm), multiplied by 100. The attained DD% of 100 ± 1 means that an average of one methacrylic group was introduced in every repeating unit of the polymer. This is an unusual way to express the DD%, which is more typically calculated as the fraction of hydroxyl groups per repeating unit that have been methacrylated [46]. Following the classic definition of DD%, the synthesized GG-MA has a derivatization degree of 10 ± 1%, which means that only one hydroxyl group of the repeating unit was modified. However, we found the novel proposed DD% to be a more intuitive and simple way to define the methacrylation degree of GG-MA.

**Figure 1:**
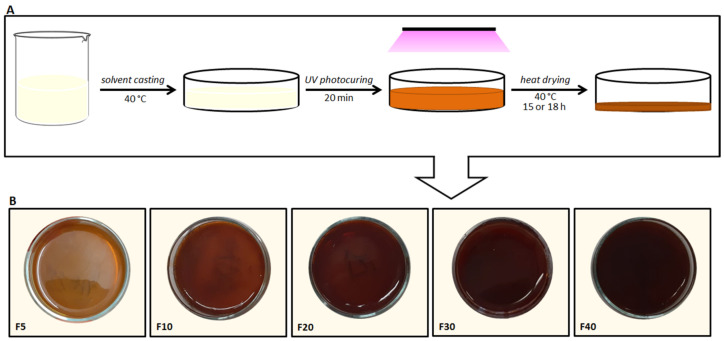
(**A**) Schematic of the procedure followed for the preparation of the nanocomposite films. (**B**) Images of the films obtained after the photocuring step.

**Figure 2:**
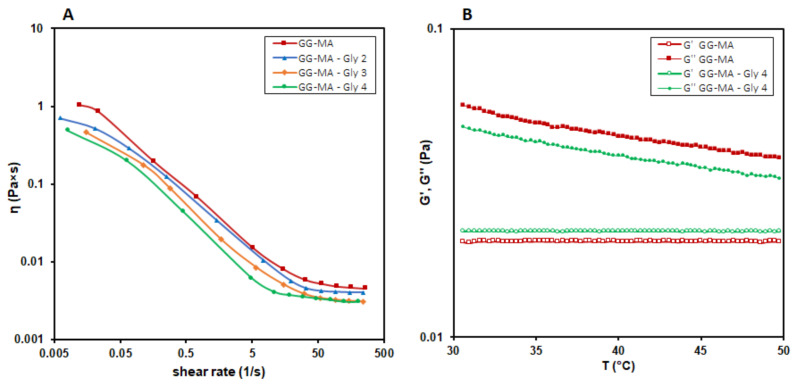
Effect of Gly on (**A**) the flow properties and (**B**) the sol-gel transition of GG-MA solutions (2% *w/v*).

The obtained GG-MA was used to prepare nanocomposite films, adopting a solvent casting procedure coupled with UV photocuring and subsequent heat drying, as schematized in Figure 1A. The film formulation was optimized with a trial-and-error approach. Specifically, the casting and spreadability of the film-forming solutions as well as flexibility, elasticity, peeling and uniformity of the films were the main attributes evaluated for the optimization of the formulations. The first attempts to prepare GG-MA films were made considering our previous studies on GG-based OTFs (Oral Thin Films) [40,43]. For this reason, a 2.0% *w/v* polymer concentration was used in samples A, B and C (Table 1), which show a typical pseudoplastic behavior and viscosity values at 40.0 ± 0.1 °C, suitable for the casting process (Figure 2A). GG-MA films were prepared using Gly as a plasticizer, which is essential to easily peel off film from the support and allow for the formation of films with adequate elasticity, flexibility and strength, as observed with previous GG-based OTFs. Indeed, using Gly as the plasticizing agent, the mechanical properties of GG-based OTFs were significantly improved [40,43]. For these reasons, Gly was added to GG-MA solution, and its influence on the viscosity of the film-forming solution was investigated. Indeed, the plasticizer may affect the flow properties of the polymer solutions, which are of fundamental importance when polymeric films are prepared using the solvent casting technique, since inadequate viscosity and viscoelasticity may affect the final outcome, leading to barely uniform and homogenous films. The flow curves of solutions containing 2.0% *w/v* of GG-MA and different concentrations of Gly, from 2.0% to 4.0% *w/v*, are reported in Figure 2A. Regardless of the amount of plasticizer used, the solutions had almost the same behavior, with viscosity values suitable for casting in all the cases. In addition, further rheological studies were carried out, aimed at evaluating the physical state and internal structure of the polymer system (physical gel or solution) during the casting process. For this reason, temperature sweep tests were recorded in the temperature range from 30.0 to 50.0 °C on GG-MA at 2.0% *w/v,* alone or mixed with 4% *w/v* of Gly (Figure 2B). It was observed that, regardless of the presence of the plasticizer, the cross-over of the G’ and G” moduli did not occur in the temperature range considered. Therefore, the entire casting process and the following photocuring step could be carried out on polymer solutions, which allows for adequate polymer chain mobility and the formation of homogeneous and uniform films. The early sol-gel transition and the consequent formation of a physical gel would result in the uneven spreading of the mixtures on the surface of the support during the casting process. Moreover, it may reduce the polymer mobility and interfere with the following free radical crosslinking of GG-MA during the UV irradiation step. On the basis of these results, 2.0% and 3.0% *w/v* were selected as the starting concentrations of GG-MA and Gly, respectively, and 40 °C was selected as the casting temperature. The total volume of casted solution was fixed to 6 mL in order to obtain films of suitable thickness after the drying step, carried out at 40 °C for 15 h. Different volumes of AgNO_3_ (30% *w/v*) were added to the polymer solution in order to obtain increasing concentrations of silver ions and consequently AgNPs (see samples A_5_–A_50_ in Table 1).

The results showed that the use of AgNO_3_ concentrations higher than 40 mg/mL gave rise to the formation of a polymer mixture unsuitable for solvent casting, owing to an evident increase in the viscosity, which resulted in inhomogeneous and irregular deposition of the film-forming mixture on the film mold. Therefore, 40 mg/mL was established as the maximum concentration of AgNO_3_. After casting, the polymer mixtures were UV irradiated for 20 min in the presence of the photoinitiator Irgacure 2959. The photocured mixtures were then heat dried at 40 °C for 15 h, and the obtained films were peeled off from the glass support and visually observed. All the obtained films showed an evident and marked tendency to shrink at the end of the drying process; this phenomenon was less evident in samples with lower AgNPs content. Therefore, to limit this film defect and, at the same time, improve film handling, further films were prepared, varying the concentrations of GG-MA and Gly (samples B_40_, C_40_, D_40_, E_40_ in Table 1). In these cases, it was observed that increasing Gly from 3.0 to 4.0% *w/v* still resulted in evident film shrinkage, whereas the characteristics of the film were considerably improved when the concentration of Gly was reduced from 3.0 to 2.0% *w/v*. The appearance of the films and the tendency to shrink were further improved when the concentration of GG-MA was reduced from 2.0 to 1.5 *w/v* (samples D_40_ and E_40_ in Table 1). The rheological analyses (flow-sweep measurements and oscillatory temperature-sweeps analyses) were not repeated on these samples, since the polymer concentration was reduced to 1.5% *w/v*, and it was reasonable to assume adequate rheological properties for casting in these cases as well. Both D_40_ and E_40_ films did not shrink markedly and almost preserved the diameter of the glass support. At the same time, these films showed consistency and flexibility suitable for their possible application as wound dressings, particularly those obtained from GG-MA 1.5% and Gly 1.0% *w/v* mixtures. These concentrations were therefore employed for the preparation of films with different AgNPs content. However, when mixed with AgNO_3_, the film-forming solutions still showed inappropriate rheological properties for even casting on the glass support. To avoid these problems, the weight ratio between GG-MA and AgNO_3_ was half reduced, and a double volume of the film-forming mixture (passing from 6 mL to 12 mL) was casted on the support. In this way, the formation of homogeneous and uniform films was possible, reaching a concentration of AgNO_3_ as high as 40 mg/mL. Obviously, by increasing the final volume of the film-forming solution, it was necessary to further optimize the duration of the drying step. In particular, the drying time was prolonged to 18 h in order to obtain finished films with optimal residual moisture content, which is fundamental for proper film handling. Finally, nanocomposite films with different AgNPs content (samples F_5_, F_10_, F_20_, F_30_, F_40_ in Table 1) could be obtained using five different AgNO_3_ concentrations (5, 10, 20, 30 and 40 mg/mL), GG-MA at 1.5% *w/v*, Gly at 1.0% *w/v* and casting a final volume of 12 mL. Under these conditions, it was possible to produce the nanocomposite films reported in Figure 1B. Despite the high concentrations of AgNO_3_, particularly in the F_40_ formulation, the formation of crosslinked networks was not hampered but maybe assisted by silver.

### 3.2. Film Characterization

All the optimized films were characterized for the thickness and the tensile properties, and the results are reported in Table 2.

The thickness was measured in six different points of each film to assess their homogeneity and uniformity. The obtained results showed the formation of films having almost uniform thickness, thus confirming the suitability of the set-up conditions adopted for the casting and drying processes. Moreover, a progressive increase in the thickness values of the films can be observed as the GG-MA/AgNO_3_ weight ratio decreases, as expected.

The tensile properties of the films were also evaluated, and the resulting tensile modulus and strength values are reported in Table 2. A stiffening and strengthening effect for increasing AgNPs content can be observed—in particular, an increase of 61% in tensile modulus and of 48% in tensile strength is achieved moving from F_5_ to F_40_ formulation. Standard deviation values highlight a variation in the range of 5–15% from the mean value, highlighting the good repeatability in the tensile properties. These results are coherent with the ones reported by Thiagamani et al. [47] for banana peel powder and AgNPs cellulose-based films and by Fan et al. [48] for cellulose nanocrystals and AgNPs PVA films.

**Figure 3:**
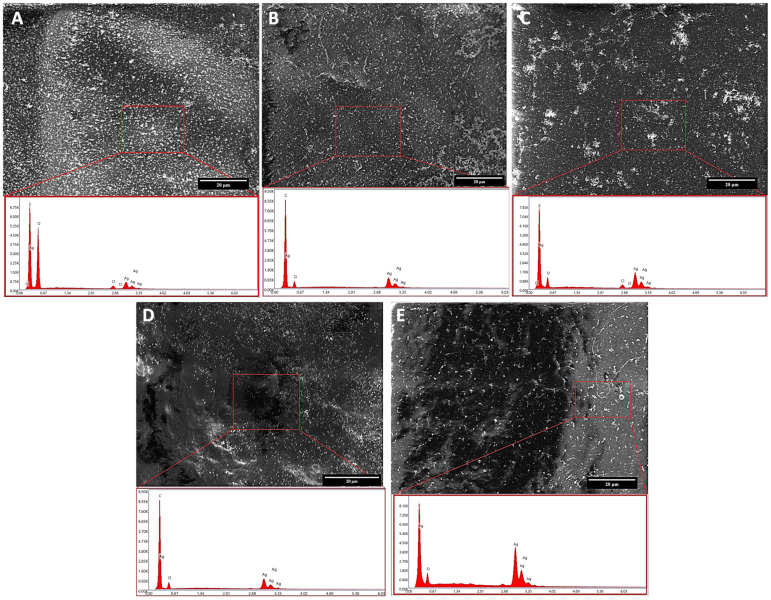
SEM micrographs and corresponding EDS analysis of (**A**) F_5_, (**B**) F_10_, (**C**) F_20_, (**D**) F_30_ and (**E**) F_40_ film, respectively. The scale bar is 20 μm.

The optimized films were also characterized for morphology and color by FE-SEM and colorimetry, respectively. The morphological and elemental analyses of all the F formulations (F_5_, F_10_, F_20_, F_30_ and F_40_) are reported in Figure 3. All the films show a smooth surface and three typical peaks, namely, the ones of carbon (C), oxygen (O) and silver (Ag). The first two peaks can be ascribed to the polymeric nature of the film being the main components of the backbone. Concerning the Ag peak deriving from the nanoparticles, it displays an increasing intensity, moving from 5 mg/mL to 40 mg/mL, thus confirming the increasing number of AgNPs dispersed in the film. Small traces of chloride can be also detected in the samples and must be ascribed to the buffer solution used to remove glycerol from the films. This procedure was necessary to achieve sample desiccation fundamental to ensuring specimen analyzability through SEM.

**Figure 4:**
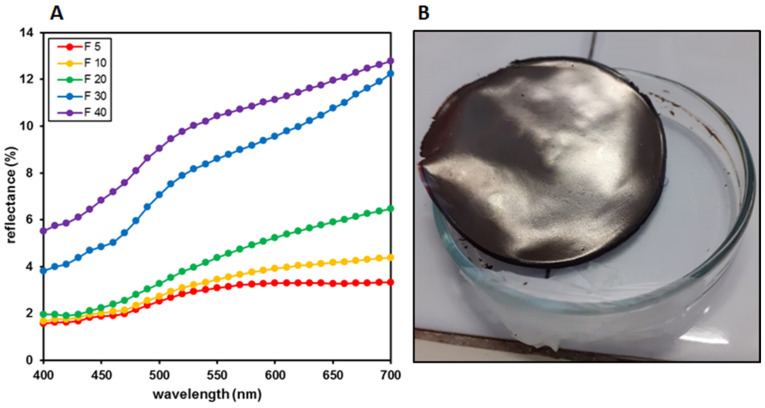
(**A**) Reflectance curves of films F_5_, F_10_, F_20_, F_30_ and F_40_. (**B**) Photograph of F_40_ film showing the mirror effect observed after the heat drying step.

Color analyses were also performed on the finished films. Figure 1B shows a uniform color distribution and a constant browning expressed by the fresh films obtained after the photocuring process, consistent with the increasing AgNPs content. On the contrary, the reflectance curves recorded on the corresponding heat dried systems showed a constant rise correlated with a constant increase in the L* values (from 20 to 35) from F_5_ to F_30_ (Figure 4A). This result is apparently at odds with the increase in the a* and b* values and the correlated color saturation, which rises from 9 to 14. In F_40_, a weak decrease of b*, reflecting on the related saturation, was observed, but a similar trend, with a further L* increase, was shown, denoting that the rise of this parameter is relatively independent of the other two parameters a* and b* (Table 3).

On the whole, this means that, notwithstanding the constant browning, which was also visually perceived on the films’ surfaces (Figure 4B), what is really expressed by the reflectance curves is the appearance of the mirror effect, which in turn is correlated with the increased silver content and its clustering in the solid phase, obtained during the heat drying process [49,50].

### 3.3. Swelling Measurements

**Figure 5:**
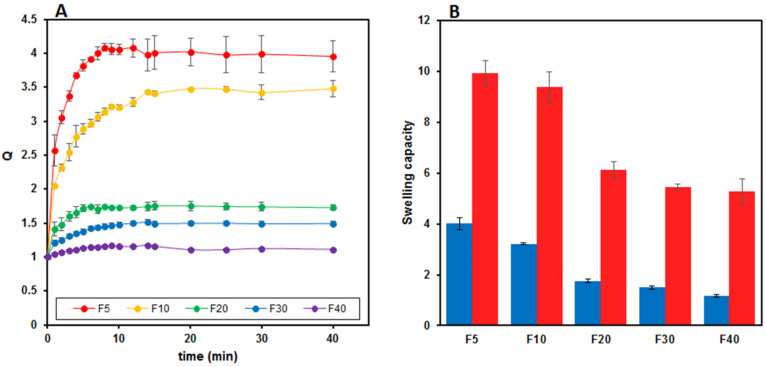
Swelling capacity of nanocomposite films measured in PB (pH = 7.4) at 37.0 ± 0.1 °C. (**A**) Dynamic swelling over time. (**B**) Comparison between the swelling degree (blue bars) and water uptake capacity (red bars) of films with different AgNPs content. Results are reported as the mean ± the standard deviation (*n* = 3).

The results obtained from the dynamic swelling studies conducted at 37.0 °C in PB (pH = 7.4) on films containing increasing AgNPs concentrations are reported in Figure 5A. A progressive decrease in the *Q* value is evident, passing from F5 to F40 film (blue data in Figure 5B). However, an evident loss of AgNPs and probably of Gly was observed during the swelling experiments. Indeed, as soon as the films came into contact with the swelling medium, an outward diffusion of AgNPs took place. It is likely that Gly was also involved in the diffusion process and lost from the films, as already reported for GG-based OTFs [40]. Therefore, the *Q* value, typically calculated as the ratio between the swollen and dry weight of the film, is not ideal to describe the swelling process of these films. If, on the one hand, the film adsorbs PB going into swelling, on the other hand, some film components diffuse toward the external medium and are lost from the film. Therefore, the obtained *Q* values are not very reliable and, for this reason, they were corrected, taking into account Gly and silver loss. To this end, a series of swelling experiments were carried out, limiting to 15 min the contact time of the films with the swelling medium. After this time, each system was recovered, dried and weighed to estimate the entity of weight loss dependent on the outward diffusion of Gly and AgNPs. The measured weight values (*W_e_*) confirmed that, during the swelling process, an effective decrease in the weight of the film sample took place. Moreover, using these *W_e_* values, the swelling degree of the films was recalculated and expressed as water uptake (*WU*). The obtained results are reported in Figure 5B (red data). It can be observed that, in any case, even taking into account the loss of AgNPs and Gly that occurred during the swelling process, the *Q* and *WU* values follow the same decreasing trend. Indeed, the water uptake capacity of the films also decreases with increasing AgNPs content, and this behavior may be explained by the hydrophobic nature of the AgNPs or, alternatively, by a silver-mediated crosslinking of GG-MA. It may be likely that silver is also involved in the network formation such that more silver means a denser crosslinked structure and consequently a lower swelling degree. Therefore, even if some components of the films are lost during the swelling process, the AgNPs still present inside the films influence and limit the swelling capacity of the system. While both the *Q* and *WU* parameters can be used to describe the swelling ability of these nanocomposite films, the *WU* values are, in our opinion, more reliable and allow for the better assessment of the behavior of these systems when in contact with a wound, particularly an exudative one.

### 3.4. Release Studies

**Figure 6:**
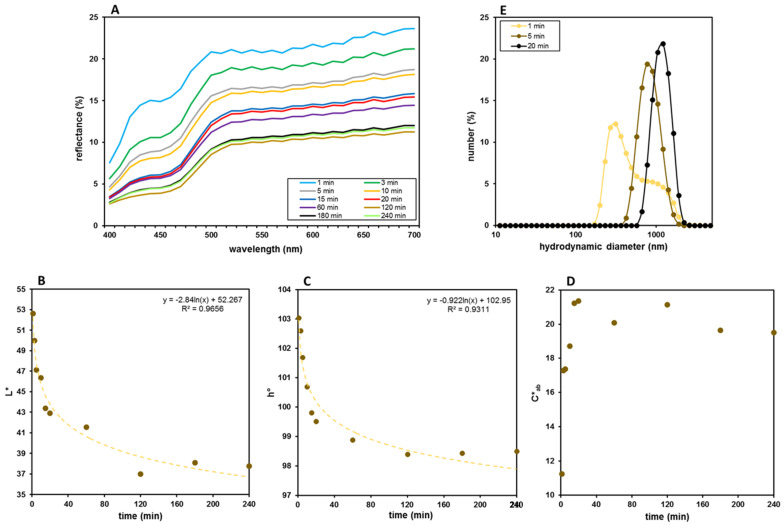
Release of AgNPs in PB (pH = 7.4) from F_40_ films evaluated by colorimetric and DLS analyses of the release medium over time. (**A**) Reflectance curves recorded at different time points. Trends of (**B**) L*, (**C**) h° and (**D**) C*_ab_ values. (**E**) Hydrodynamic diameter of released AgNPs measured at three different and representative time points.

The release of AgNPs was studied in PB (pH = 7.4) at 37.0 ± 0.1 °C and monitored performing color and DLS analyses on samples of the release medium taken at different times. The results reported in Figure 6A show a constant browning correlated with a smooth lowering of the reflectance curves. Differently from the logarithmic trends shown by the lightness and the tonality (Figure 6B,C), the color saturation (C*_ab_) reaches a maximum between 15 min and 20 min, followed by a substantial stabilization at slightly lower values (Figure 6D). Considered on the whole, the color data of the release medium seem to indicate that these nanocomposite films could be adaptable to a slow and prolonged release of AgNPs and could potentially represent a good and interesting system to maintain a constant silver content in the wound. DLS measurements evidenced a progressive and almost rapid increase in the hydrodynamic diameter of AgNPs in the release medium (Figure 6E). Therefore, quantitative information could hardly be obtained from colorimetric data, because color parameters are influenced by the number of AgNPs released over time but also by their size [51,52].

### 3.5. Antimicrobial Activity of Films Loaded with AgNPs

To verify the possible application of the GG-MA films loaded with AgNPs as dressings for the treatment of infected wounds, antimicrobial activity assays were carried out in solid and liquid culture media.

#### 3.5.1. Antimicrobial Activity in Solid Culture Media (Diffusion Test)

The halos formed after 24 h of incubation of the double layer plates were all of a few millimeters of diameter (c.a. 5 mm), independently from the Ag concentration in the GG-MA based film. Indeed, the halos were the same for all Ag concentrations assayed, probably because the silver contained in the disks did not diffuse into the soil. It is possible that the agar medium was not sufficiently hydrated to allow for adequate AgNPs diffusion. This type of assay does not allow for the proper assaying of the antimicrobial activity of polymeric films; therefore, further studies were carried out working under experimental conditions capable of better allowing the release of AgNPs.

#### 3.5.2. Antimicrobial Activity in Liquid Culture Media

**Figure 7:**
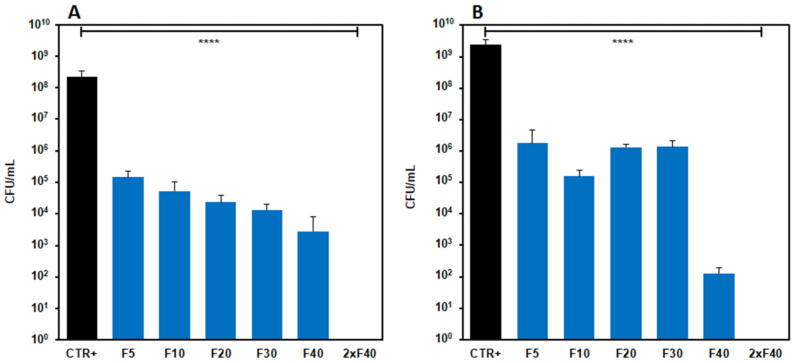
Antimicrobial activity of nanocomposite films tested in liquid culture media on *Staphylococcus aureus* ATCC 6538 and *Escherichia coli* MG1655. Liquid culture media: (**A**) *S. aureus*, (**B**) *E. coli*. Results are reported as the mean ± the standard deviation (*n* = 3). **** *p* < 0.0001.

**Figure 8:**
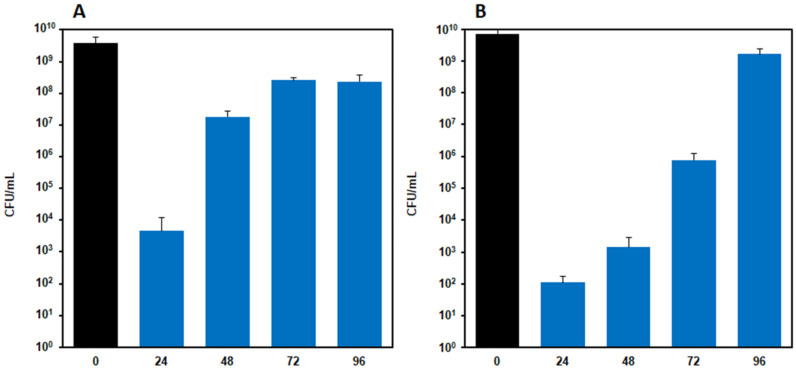
Antimicrobial activity of F40 nanocomposite films tested in liquid culture media on (**A**) *Staphylococcus aureus* ATCC 6538 and (**B**) *Escherichia coli* MG1655. Films were daily moved in fresh culture media containing 10^8^ CFU/mL microorganisms for 4 days, and, at each time point (24, 48, 72 and 96 h), the residual bacterial concentration was measured. Results are reported as the mean ± the standard deviation (*n* = 3).

The evaluation of the antimicrobial activity of films with AgNPs carried out in liquid culture media showed that, for both strains, after 24 h of incubation, the concentration of bacteria significantly decreased with respect to the control (*p* < 0.0001) for each Ag concentration assayed. The bacteria concentration decreased with the increasing of AgNPs concentrations in the film, particularly with *S. aureus* (Figure 7A). On the contrary, similar effects were produced on the *E. coli* strain by formulations from F_5_ to F_30_, whereas the F_40_ film caused a marked reduction of bacteria concentration. Indeed, the oscillations observed for this strain were repeated in all the experiments performed, and the observed differences among the F_5,_ F_10_, F_20_ and F_30_ formulations were not significant, whereas significant differences were observed between the F_30_ and F_40_ films. Furthermore, the highest Ag concentration tested (80 mg/mL, obtained using two samples of F_40_ film) turned out to be bactericidal for both the strains. Further studies were carried out on F_40_ nanocomposites films to test their antibacterial activity over time. To this end, the F_40_ films were challenged daily with 10^8^ microorganisms for 4 days [53]. The results reported in Figure 8 show that F_40_ films possess substantial antibacterial potential, particularly against *E. coli*. Maximum activity is observed at day 1 due to the burst release of silver; however, even at days 2 and 3, the nanocomposite film is still able to reduce the initial number of microorganisms by at least four log fold. The level of contamination of infected wounds is not always easily predictable; however, it can be safely considered that the challenge of 10^8^ microorganisms per day for 4 days would be almost unrealistic in a real wound, unless it was grossly reinfected. This test allowed for the maximization of the work that the dressing must do in order to gauge its remaining antimicrobial capacity.

Results denote that Ag still maintain a good antimicrobial activity versus the two bacterial strains assessed, even when loaded on GG-MA based film, indicating this delivery system to be a good candidate for the treatment of wound infections often difficult to eradicate.

Globally, the obtained results open prospective for the application of photocurable GG derivatives for the fabrication of nanocomposite films with antibacterial properties. GG is only marginally considered in the scenario of the wound dressing materials [39], even if it possesses interesting features to efficiently support wound healing [54,55,56,57].

## 4. Conclusions

Nanocomposite films containing AgNPs were produced through the reduction of Ag^+^ to Ag^0^ and clustering into nanostructures within polymer networks under UV irradiation. This approach allowed, after casting of the film-forming solution, for the concurrent formation of AgNPs and chemical crosslinking of the photocurable polymer GG-MA. The proposed procedure represents an interesting and safe alternative for silver dressing fabrication.

GG-MA/silver ions and GG-MA/plasticizer weight ratios resulted in the most critical experimental parameters for the formation of homogeneous and uniform nanocomposite films with adequate mechanical properties. In particular, the GG-MA/Ag^+^ weight ratio affected the rheological properties of the film-forming mixture, which critically influence the solvent casting process and the even distribution of the mixture on the support. Instead, the GG-MA/Gly weight ratio affected the shrinkage of the film after heat drying. Therefore, both these parameters were finely optimized for the formation of homogeneous, uniform and resistant films containing increasing concentrations of AgNPs, as evaluated by SEM observation and tensile tests. The presence of the nanoparticles modestly influenced the mechanical properties of the final films, whereas it significantly affected their swelling capacity. Specifically, increasing concentrations of AgNPs determined a progressive decrease in the swelling ability of the films, even considering the partial loss of film components (plasticizer and AgNPs) during the swelling studies. The release of AgNPs was qualitatively investigated by colorimetric analysis, which revealed the ability of the films to provide sustained release of AgNPs over time. The outward diffusion of AgNPs was fundamental for the antibacterial activity of the films when tested on *Staphylococcus aureus* and *Escherichia coli.* Indeed, the proposed films showed the capacity to significantly decrease the bacteria concentration with respect to the control. Globally, the proposed approach allows for the development of nanocomposite films with a facile and safe synthesis procedure, which avoids the use of toxic reagents for silver reduction. The obtained films showed interesting features and have potential application as innovative dressing materials for infected wound management.

## Figures and Tables

**Table 1:** Composition of the film-forming mixtures for film preparation. Film samples were labelled with letters (the same letter was used for samples obtained from film-forming mixtures with the same concentrations of GG-MA and Gly) and a subscript, which was used to specify the amount of silver salt included in the formulation.

Sample	GG-MA (% *w*/*v*)	Gly (% *w*/*v*)	AgNO_3_ (mg/mL)	Casted Volume (mL)
A_5_	2.0	3.0	5	6
A_10_	2.0	3.0	10	6
A_20_	2.0	3.0	20	6
A_30_	2.0	3.0	30	6
A_40_	2.0	3.0	40	6
A_50_	2.0	3.0	50	6
B_40_	2.0	4.0	40	6
C_40_	2.0	2.0	40	6
D_40_	1.5	2.0	40	6
E_40_	1.5	1.0	40	6
F_5_	1.5	1.0	5	12
F_10_	1.5	1.0	10	12
F_20_	1.5	1.0	20	12
F_30_	1.5	1.0	30	12
F_40_	1.5	1.0	40	12

**Table 2:** Thickness, tensile modulus and tensile strength of films F_5_, F_10_, F_20_, F_30_ and F_40_. The measurements were carried out at least in triplicate, and the results are reported as mean values ± standard deviation.

Sample	Thickness (μm)	Tensile Modulus (MPa)	Tensile Strength (MPa)
F_5_	72.2 ± 2.6	2.13 ± 0.24	0.41 ± 0.08
F_10_	97.0 ± 6.4	2.70 ± 0.28	0.45 ± 0.07
F_20_	116.0 ± 9.0	3.05 ± 0.24	0.47 ± 0.06
F_30_	130.8 ± 6.4	3.21 ± 0.17	0.59 ± 0.03
F_40_	152.3 ± 10.6	3.43 ± 0.30	0.61 ± 0.04

**Table 3:** Colorimetric parameters of heat dried films F_5_, F_10_, F_20_, F_30_ and F_40_.

Film Samples	L*	a*	b*	C*_ab_	h°
F_5_	19.68	−0.02	8.62	8.62	91.34
F_10_	21.39	0.02	9.88	9.95	83.04
F_20_	24.34	1.02	12.78	13.03	78.75
F_30_	34.83	1.21	13.88	13.91	85.72
F_40_	38.16	2.54	11.10	11.11	89.85

## Data Availability

The data presented in this study are available on request to the corresponding authors.

## Sample Availability

Samples of the compounds are available from the corresponding authors.
